# Choosing and using diversity indices: insights for ecological applications from the German Biodiversity Exploratories

**DOI:** 10.1002/ece3.1155

**Published:** 2014-08-28

**Authors:** E Kathryn Morris, Tancredi Caruso, François Buscot, Markus Fischer, Christine Hancock, Tanja S Maier, Torsten Meiners, Caroline Müller, Elisabeth Obermaier, Daniel Prati, Stephanie A Socher, Ilja Sonnemann, Nicole Wäschke, Tesfaye Wubet, Susanne Wurst, Matthias C Rillig

**Affiliations:** 1 Institute of Biology, Dahlem Center of Plant Sciences, Freie Universität BerlinAltensteinstr 6, Berlin, 14195, Germany; 2 Department of Biology, Xavier University3800 Victory Parkway, Cincinnati, Ohio, 45207; 3 School of Biological Sciences, Queen’s University Belfast97 Lisburn Road, Belfast, BT9 7BL, Northern Ireland; 4 Department of Soil Ecology, UFZ- Helmholtz Centre for Environmental ResearchTheodor-Lieser-Strasse 4, Halle/Saale, 06120, Germany; 5 Institute of Biology, University of LeipzigJohannisallee 21-23, Leipzig, 04103, Germany; 6 German Centre for Integrative Biodiversity Research (iDiv)Deutscher Platz 5e, Leipzig, 04103, Germany; 7 Institute of Plant Sciences, University of BernAltenbergrain 21, Bern, 3013, Switzerland; 8 Department of Animal Ecology and Tropical Biology, University of WürzburgAm Hubland, Würzburg, 97074, Germany; 9 Department of Chemical Ecology, Bielefeld UniversityUniversitätsstr. 25, Bielefeld, 33615, Germany; 10 Institute of Biology, Applied Zoology/Animal Ecology, Freie Universität BerlinHarderslebener Strasse 9, Berlin, 12163, Germany; 11 Berlin-Brandenburg Institute of Advanced Biodiversity Research (BBIB)Altensteinstr 6, Berlin, 14195, Germany

**Keywords:** Arbuscular mycorrhizal fungi, arthropods, Berger–Parker, chemical diversity, Hill’s powers, molecular diversity, plant diversity, *Plantago lanceolata*, Shannon index, Simpson’s index

## Abstract

Biodiversity, a multidimensional property of natural systems, is difficult to quantify partly because of the multitude of indices proposed for this purpose. Indices aim to describe general properties of communities that allow us to compare different regions, taxa, and trophic levels. Therefore, they are of fundamental importance for environmental monitoring and conservation, although there is no consensus about which indices are more appropriate and informative. We tested several common diversity indices in a range of simple to complex statistical analyses in order to determine whether some were better suited for certain analyses than others. We used data collected around the focal plant *Plantago lanceolata* on 60 temperate grassland plots embedded in an agricultural landscape to explore relationships between the common diversity indices of species richness (S), Shannon’s diversity (H’), Simpson’s diversity (D_1_), Simpson’s dominance (D_2_), Simpson’s evenness (E), and Berger–Parker dominance (BP). We calculated each of these indices for herbaceous plants, arbuscular mycorrhizal fungi, aboveground arthropods, belowground insect larvae, and *P. lanceolata* molecular and chemical diversity. Including these trait-based measures of diversity allowed us to test whether or not they behaved similarly to the better studied species diversity. We used path analysis to determine whether compound indices detected more relationships between diversities of different organisms and traits than more basic indices. In the path models, more paths were significant when using H’, even though all models except that with E were equally reliable. This demonstrates that while common diversity indices may appear interchangeable in simple analyses, when considering complex interactions, the choice of index can profoundly alter the interpretation of results. Data mining in order to identify the index producing the most significant results should be avoided, but simultaneously considering analyses using multiple indices can provide greater insight into the interactions in a system.

## Introduction

Biodiversity represents the variety and heterogeneity of organisms or traits at all levels of the hierarchy of life, from molecules to ecosystems. Typically, the focus is on species diversity, but other forms of diversity, such as genetic and chemical diversity, are also important and informative. Even after deciding which form of diversity to measure, quantifying biodiversity remains problematic because there is no single index that adequately summarizes the concept (Hurlbert 1971; Purvis and Hector 2000). Richness (S), or the number of species or attributes present, is the simplest metric used to represent diversity (Whittaker 1972), and it remains the most commonly applied (Magurran 2004). Intuitively, species or trait abundance is also important for diversity, and the proportional abundance of species can be incorporated into indices representing diversity. The simplest of these indices was proposed by Berger and Parker, has an analytical relationship with the geometric series of the species abundance model (May 1975; Caruso et al. 2007), and reports the proportional abundance of only the most abundant species in the population (BP, Table 1, Berger and Parker 1970).

**Table 1:** Formulas used to calculate diversity measures analyzed

Metric	Traditional formula^1^	Surrogate in Hill’s Series, Hill’s power^2^
Richness (S)	Number of species	S, 0
Shannon’s diversity (H’)	−∑*P*_i_ ln(*P*_i_)	exp(H’), 1
Simpson’s diversity (D_1_)	1 − ∑ 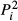	D_2_, 2
Simpson’s dominance (D_2_)	1/∑ 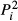	D_2_, 2
Berger–Parker dominance (BP)	*P*_max_	BP^−1^, ∞
Simpson’s evenness (E)	D_2_/S	–

1 *p*_i_ is the proportion of individuals belonging to species *i*; *p*_max_ is the proportion of individuals belonging to the most abundant species. Formulas from McCune and Grace (2002), Shannon (1948), and Simpson (1949).

2 Formulas from Hill (1973).

There have been numerous attempts to create compound indices that combine measures of richness and abundance. Foremost among these are the Shannon’s diversity (H’) and Simpson’s diversity (D_1_) indices (Table 1), which differ in their theoretical foundation and interpretation (Magurran 2004). H’ has its foundations in information theory and represents the uncertainty about the identity of an unknown individual. In a highly diverse (and evenly distributed) system, an unknown individual could belong to any species, leading to a high uncertainty in predictions of its identity. In a less diverse system dominated by one or a few species, it is easier to predict the identity of unknown individuals and there is less uncertainty in the system (Shannon 1948). This metric is common in the ecological literature, despite its abstract conceptualization (Magurran 2004). D_1_ is the complement of Simpson’s original index and represents the probability that two randomly chosen individuals belong to different species (McCune and Grace 2002). D_2_ is closely related to D_1_, being the inverse of Simpson’s original index (Simpson 1949). Both of these transformations serve to make the index increase as diversity intuitively increases, and although both are used, D_2_ is more common (Magurran 2004).

Finally, evenness represents the degree to which individuals are split among species with low values indicating that one or a few species dominate, and high values indicating that relatively equal numbers of individuals belong to each species. Evenness is not calculated independently, but rather is derived from compound diversity measures such as H’, D_1_, and D_2_, as they inherently contain richness and evenness components. However, evenness as calculated from H’ (J’) is of only limited use predictively because it mathematically correlates with H’ (DeBenedictis 1973). E, calculated from D_2_ (Table 1), is mathematically independent of D_1_ (Smith and Wilson 1996) and therefore a more useful measure of evenness in many contexts.

**Figure 1:**
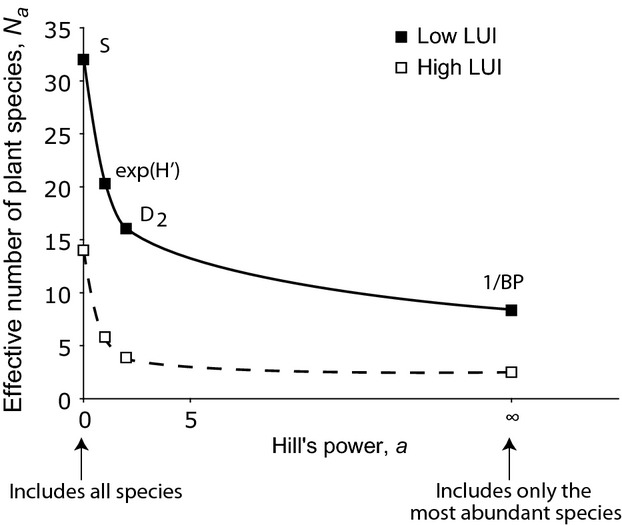
Herbaceous plant diversity in sites with representative high and low land use intensity (LUI). The low LUI site (AEG07) was an unfertilized sheep pasture, while the high LUI site (AEG02) was a fertilized meadow that was mown three times a year. The effective species number decreases in both sites as Hill’s power increases and increasingly abundant species are excluded.

Strong correlations between diversity measures should not be surprising as they represent aspects of the same phenomenon. In fact, most of the measures analyzed here can be derived from the same basic generalized entropy formula *N*_*a*_ = (∑
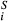
_= 1_ *P*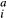)^1/(1−*a*)^, where *N*_*a*_ is the effective species number, *S* is total species number, *P*_*i*_^*a*^ is the proportional abundance of species *i*, and *a* is the power (Table 1; Hill 1973). H’ is equally sensitive to rare and abundant species; sensitivity to rare species increases as *a* decreases from 1, and sensitivity to abundant species increases as *a* increases from 1 (Fig. 1; Jost 2007). Therefore, S is sensitive to rare species, D_1_ and D_2_ are sensitive to abundant species, and BP is sensitive to only the most abundant species. As all the *N*_*a*_’s have species as the unit, the range of values can be interpreted as a continuum from effective number of the most rare species to effective number of the most abundant species.

Despite the strong relationships between these diversity measures, they are not interchangeable and there has been much debate over which is appropriate in various contexts. For purposes where ranking sites by their level of diversity is the primary goal, such as in conservation planning when selecting sites to be protected, compound indices are often preferred over species richness (Magurran and Dornelas 2010). This was true for macroinvertebrate diversity in riverine sites and for plant diversity in temperate grasslands, where H’ and D_2_ were calculated, respectively, and found to discriminate sites more effectively than S (Wilsey et al. 2005; Heino et al. 2008). In contrast, Magurran and Dornelas (2010) argue against using compound indices when the objective is to detect effects of external factors on diversity, such as when assessing anthropogenic impacts on the environment. There is some empirical evidence that simple indices are indeed more effective in these cases, as S correlated better with landscape parameters than either J’ or H’ for aquatic macroinvertebrates (Heino et al. 2008). Leinster and Cobbold (2012) advocate for presenting diversity as a continuous metric analogous to Hill’s series (Hill 1973). This approach is useful when the change in diversity itself is of interest. However, as experiments and field surveys become ever more complex, an increasingly common objective in biodiversity studies is an understanding of interactions between diversities (i.e., how changes in biodiversity of one trophic level affect biodiversity of other trophic levels). In analyses such as these, where path models can provide great insight into interactions, a single measure of diversity is needed. Additional insights into community dynamics can be obtained by including trait-based diversity measures. For example, when modeling changes in species diversity throughout a community, knowledge of the genetic and chemical diversity of the primary producer (e.g., a plant) in the system would provide mechanistic insights into any changes in herbivorous insect diversity that could be related to the complexity of herbivore defenses or attractants displayed by the plant. It is unclear which diversity index is most effective at this type of complex community level analysis.

We attempted to clarify these complex relationships, and develop guidelines for practical applications, using data collected in grasslands throughout Germany as part of the Biodiversity Exploratories research network, which consists of 150 plots in three regions that are managed with combinations of fertilization, mowing, and grazing (Fischer et al. 2010). We focused on 60 plots containing *Plantago lanceolata*, and collected data around focal *P. lanceolata* plants in each plot. Focusing data collection around one plant species allowed us to collect in depth data on the dynamics of similarly structured communities spread across a land use gradient. In addition to species diversity of the plants, arbuscular mycorrhizal fungi (AMF), aboveground arthropods, and belowground insect larvae, we also measured neutral molecular and chemical diversity of *P. lanceolata*. These neutral measures have not yet been included in analyses of this type, which have to date focused on species diversity components of biodiversity. Including them will allow us to determine whether changes in species diversity dynamics are reflected in other traits that also contribute to biodiversity.

We set out to determine how the detection and interpretation of community dynamics depended on the diversity index chosen. Community dynamics, or interactions between species, can be modeled using path analysis to describe direct and indirect interactions between species and to quantify the strength of these interactions. Including trait-based measures of diversity will provide insights into the mechanisms behind species interactions. The significance and strength of such interactions likely depends on the index used to represent diversity because the diversity indices differ in their emphasis on rare and abundant species, which are predicted to interact in different ways. We also verified that our data set agreed with earlier conclusions that compound diversity indices outperform other indices at discriminating sites as they contain information on both richness and abundance, and that simple indices giving greater weight to rare species outperform compound indices when detecting effects of disturbance on diversity. This is the first analysis to compare performance of diversity indices when quantifying diversity of multiple taxa, genetic diversity, chemical diversity, and the relationships between them. We provide guidelines for appropriate use and interpretation of diversity indices in future studies exploring biodiversity and community dynamics, which are of direct relevance to managers in terms of recommending which biodiversity measurement tools to employ.

## Materials and Methods

### Field sites, measurements, and land use index

We sampled in 60 grassland plots spread across the three regions (Schorfheide Chorin, Hainich Dün, and Schwäbische Alb) of the German Biodiversity Exploratories (see Supporting Information for a list of sites, and Fischer et al. 2010 for site details). Ten focal *P. lanceolata* plants were marked on each plot in June and July of 2008, and future sampling was conducted around these focal plants. Interactions between plants, symbiotic fungi, above and belowground herbivores, and parasitoids in temperate grasslands are extraordinarily complex. Collecting data around the same plant species on each plot allowed us to focus on a more manageable network of interactions and to explore mechanisms driving interactions by including trait-based measures of diversity. *P. lanceolata* was chosen as the focal plant because of its relative abundance in all three exploratories and because of its potential for mediating interesting interactions within and between aboveground (tritrophic interactions involving herbivores and parasitoids) and belowground biota (involving arbuscular mycorrhizal fungi and insect larvae; Gange and West 1994; Wurst and van der Putten 2007). Furthermore, some target metabolites of *P. lanceolata* are well characterized (Fontana et al. 2009), and our expansion of this knowledge base using metabolic fingerprinting approaches allowed us to investigate how chemical diversity relates to diversity of other organism groups. Finally, *P. lanceolata* is known to exhibit genetic differentiation at the population level (Kuiper and Bos 1992).

Detailed methods used to assess diversity of all organisms/traits are given in the Supporting Information. Briefly, we quantified herbaceous plant diversity by estimating percent cover of each species in a 15 cm sampling radius around the focal plants. Arbuscular mycorrhizal fungal diversity was quantified using terminal restriction fragment length polymorphism analysis of DNA extracted from rhizosphere soil of focal plants (Morris et al. 2013). Aboveground arthropods were collected from plant surfaces, and belowground insect larvae were sorted by hand or heat extracted from soil cores collected beneath focal plants. Plant molecular diversity was quantified for five loci, and chemical diversity of *P. lanceolata* was assessed by UHPLC-TOF-MS using metabolic fingerprinting techniques.

Land use intensity (LUI) on each site was quantified as an index incorporating three equally weighted variables: fertilization, mowing, and grazing intensity. For each experimental plot *i*, land use intensity LUI[*i*] is defined as the sum of each variable divided by its mean over all experimental plots per exploratory:

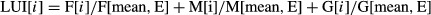


where F[*i*] is the fertilization level (kg nitrogen ha^−1^·year^−1^), M[*i*] is the frequency of mowing per year, and G[*i*] is the livestock density (livestock units ha^−1^·year^−1^) on each site *i*. The mean L[*i*] across the years 2006–2008 was used in this study, where F[mean, E], M[mean, E], and G[mean, E] are defined as the mean value across all 3 years. Land use intensity was square-root-transformed to improve normality and is dimensionless due to standardization by ratios. Land use data are based on interviews with farmers and landowners conducted each year by the management teams of each exploratory (Blüthgen et al. 2012).

### Statistical analyses

We calculated richness (S), Shannon’s diversity (H’), Berger–Parker dominance (BP), Simpson’s diversity (D_1_), Simpson’s dominance (D_2_), and Simpson’s evenness (E) for each organism/trait group (Table 1). For plant, aboveground arthropod, and belowground insect larva data, abundance was quantified as number of individuals. For mycorrhizal fungi, terminal restriction fragments (TRFs) were used as surrogates for species, and abundance was quantified as peak height of each TRF. Metabolites were used as surrogates for species in the chemical diversity data, and abundance was quantified as peak intensity. Microsatellite data from five loci were used as a surrogate for species with the population genetic data, and abundance was quantified as allele frequencies at each locus. Also for population genetic data, D_1_ is equal to the expected heterozygosity under Hardy–Weinberg equilibrium, whereas D_2_ is known as the effective number of alleles, both of which are commonly used measures of genetic diversity (Frankham et al. 2002). The formulas given in Table 1 are for complete populations, and the actual formulas for calculating these indices from sample data are slightly more complex (Magurran 2004). However, in practice, the difference between these two approaches is usually so small that the simpler formulas are generally acceptable (Magurran 2004). For organisms/traits where samples were taken around multiple focal plants per plot, the mean of each diversity index per plot was calculated.

In order to ensure that our estimates of S were reliable, we computed several estimates of total species/trait number based on extrapolations from species/trait accumulation curves, namely Chao 1, Jackknife 1, and Bootstrap for each organism/trait using R package ‘vegan’ and compared them with the observed total species/trait number (Magurran 2004; Oksanen et al. 2012).

#### Detecting community dynamics

**Figure 2:**
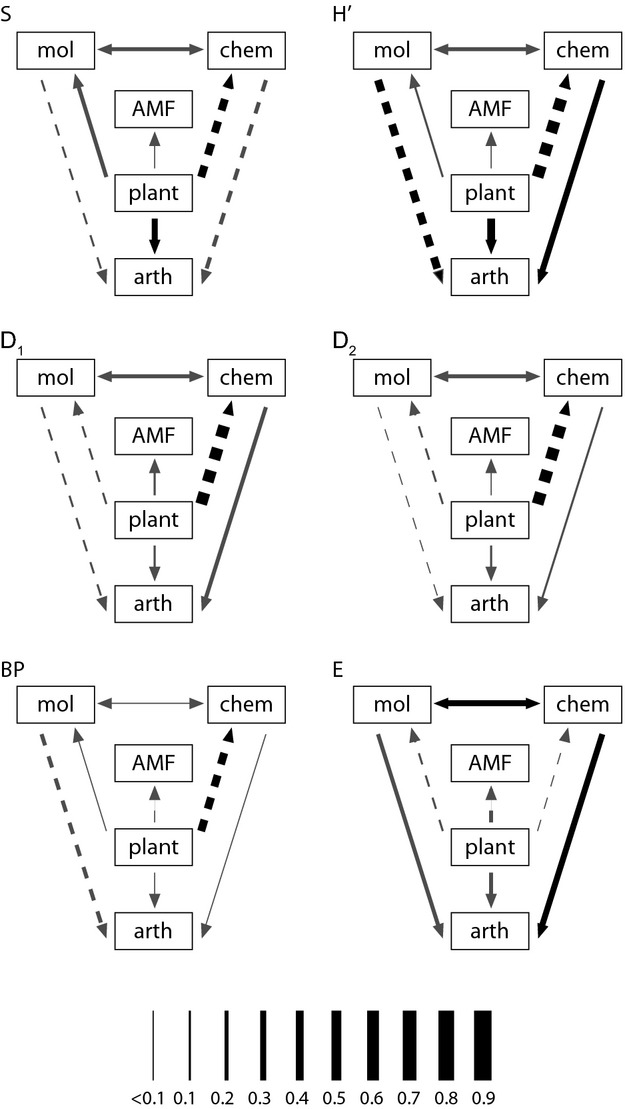
Structural equation models of links between diversity of organisms or traits measured in and around *Plantago lanceolata*. S, richness, H’, Shannon’s diversity, D_1_, Simpson’s diversity, D_2_, Simpson’s dominance, BP, Berger–Parker dominance, E, Simpson’s evenness, mol, *Plantago* molecular features, chem, *Plantago* chemical features, AMF, arbuscular mycorrhiza, plant, herbaceous plant, arth, aboveground arthropod. Solid lines indicate positive effects, while dashed lines indicate negative effects. Black lines indicate significant paths, while gray lines indicate nonsignificant paths at *α* = 0.05. The magnitude of the path coefficient is indicated by line thickness.

We constructed a path model of hypothesized relationships between organism/trait groups (Fig. 2). Belowground insect larvae were not included in the path models because they were sampled on fewer sites than other groups, and their inclusion would have reduced the sample size below acceptable limits given the complexity of our model. We ran the same structural model with each of the diversity indices, and we report model fit as chi-square and its associated *P*-value, with *P*-values greater than 0.05 indicating an acceptable fit (Hooper et al. 2008). As chi-square can be influenced by sample size, we also report the root mean square error of approximation (RMSEA), where smaller values indicate more parsimonious models, and values <0.07 suggest an adequate model fit (Hooper et al. 2008). The Tucker Lewis Non-Normed Fit Index (TLNNFI) is less sensitive to sample size and accounts for model parsimony, with values close to one indicating good model fit (Hooper et al. 2008). Path analyses were performed using the ‘SEM’ package version 0.9–16 in R (Fox 2006). All analyses were performed with R v2.11.1 and newer (R Core Team 2013).

#### Verifying earlier findings

We performed Pearson correlations between all metrics within an organism/trait group to assess relationships between the different diversity measures, after transforming data to improve normality where necessary. Each organism/trait group was analyzed separately because we could not be sure that the same pattern would be found for all groups and therefore did not want to pool data. In order to account for multiple comparisons, we used Bonferroni corrected *P*-values for all correlations within each organism/trait group. We then used principal component analysis (PCA) of correlation matrices to determine which measures of diversity were best able to differentiate sites by calculating importance values (IV) for each index (Wilsey et al. 2005). The IVs synthesize information on the importance of each principal component axis and the score for each diversity index to generate one number representing the overall importance of each diversity index in distinguishing plots based on distances between plots in the ordination.

We also performed linear regressions of each measure of diversity within organism/trait groups on LUI in order to determine whether or not the effect of land use depended on the metric chosen, after transforming data to improve normality of residuals where necessary. Indices detecting the greatest number of significant effects were judged to be the most effective, although if land use did not affect diversity in our system these indices would actually be the least effective. We used Bonferroni corrected *P-*values within each organism/trait group to account for multiple comparisons.

## Results

### Robustness of S

Estimates of total species/trait number showed that our observed richness values likely underestimated total richness for many organism/trait groups (Table 2). Estimates of aboveground arthropod, chemical, and two loci of molecular richness overlapped our observed values, suggesting that these observations are robust. For the other organism/trait groups, compound diversity measures, especially D_1_ and D_2_, may be more appropriate than S because they are not as dependent on sample size (Magurran 2004).

**Table 2:** Total observed species/order number, alleles per locus, and metabolites, and estimates of total species or trait number (mean ± SE) for each organism/trait

	Observed species number	Chao	Jackknife	Bootstrap
Plant	177	239 ± 20	240 ± 12	206 ± 6
AMF	60	71 ± 7	76 ± 4	68 ± 3
Aboveground arthropod^1^	14	16 ± 4	16 ± 1	15 ± 1
Belowground insect larvae^2^	23	30 ± 6	32 ± 3	27 ± 2
Molecular				
Locus 1	92	97 ± 4	102 ± 4	98 ± 2
Locus 2	54	59 ± 4	63 ± 3	59 ± 2
Locus 3	16	16 ± 0	16^3^	16 ± 0
Locus 4	36	39 ± 3	42 ± 2	39 ± 1
Locus 5	129	145 ± 9	153 ± 5	141 ± 3
Chemical	1449	1449 ± 0	1449^3^	1449 ± 0

1 Aboveground arthropods were identified to order.

2 Belowground insect larvae were identified to family.

3 No estimate of standard error possible because of the absence of singular alleles or metabolites.

### Detecting community dynamics

The chi-square *P*-values for the path models increased slightly in the order E ≪ S < H’ = D_1_ < D_2_ < BP (Table 3), suggesting that model fit may have improved along this gradient from indices emphasizing rare species to those emphasizing dominant species. However, the generally excellent fit of most models suggests that all adequately represent the data. The different number of significant paths in each model therefore highlights the different information emphasized by each metric. The only consistently significant path in all models (excluding E) was a negative effect of herbaceous plant diversity on chemical diversity of *P. lanceolata*, indicating that this effect was consistent across rare and abundant species. In contrast, the negative effect of plant diversity on arthropod diversity was only apparent in the S and H’ models suggesting that it is driven by rare species. No relationships appeared to be driven by changes in abundant species or traits. The path analysis also showed that E represents different information than that captured by the other diversity indices (Fig. 2, Table 3). Furthermore, the model using E fit the data poorly, while the fit of the other models was excellent, as evidenced by low RMSEA and high TLNNFI values.

**Table 3:** Model fit statistics for path analysis models

Model	Chi-squared, *P*^1^	RMSEA^2^	TLNNFI^3^
S	1.18, 0.76	<0.0001	>0.99
H’	0.51, 0.92	<0.0001	>0.99
D_1_	0.50, 0.92	<0.0001	>0.99
D_2_	0.44, 0.93	<0.0001	>0.99
BP	0.15, 0.98	<0.0001	>0.99
E	6.39, 0.09	0.14	0.35

1 For all models df = 3.

2 RMSEA – root mean square error of approximation.

3 TLNNFI – Tucker Lewis non-normed fit index.

### Verifying earlier findings

**Figure 3:**
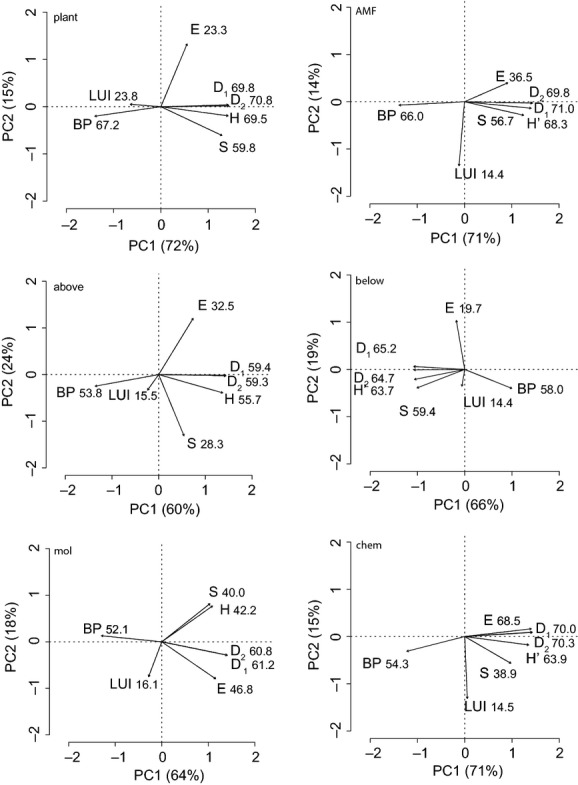
Principal component analysis of diversity measures taken in and around *Plantago lanceolata*, and land use intensity (LUI). S, richness, H’, Shannon’s diversity, D_1_, Simpson’s diversity, D_2_, Simpson’s dominance, E, Simpson’s evenness, BP, Berger–Parker dominance, plant, herbaceous plant diversity, AMF, arbuscular mycorrhizal fungal diversity, above, aboveground arthropod diversity, below, belowground insect larvae diversity, mol, *P. lanceolata* molecular diversity, chem, *P. lanceolata* chemical diversity. Numbers indicate importance values for each vector.

Correlations between diversity indices were generally strong within organism/trait groups, and BP correlated negatively with S, H’, D_1_, and D_2_ (Supporting Information, Table S1). E did not correlate in a consistent manner with any other index of diversity. For example, E was positively correlated with S for *Plantago* chemical diversity, but negatively correlated with S for aboveground arthropod diversity. Only in the belowground insect larvae did E not correlate with any other diversity index (Supporting Information, Table S1). This unpredictability of E demonstrates that it carries information not included in the other measures of diversity and argues for including E when analyzing multiple diversity indices. We used PCA to visually represent these correlations and determine if any metrics were better at differentiating plots, despite the strong correlations between all metrics (Fig. 3). Our results agreed with those of Wilsey et al. (2005) and Heino et al. (2008) showing that compound indices discriminate between plots better than more simple diversity measures.

Land use intensity generally did not affect diversity, with no evidence for stimulation or suppression of AMF, aboveground arthropod, belowground insect larvae, or *P. lanceolata* molecular or chemical diversity for any diversity index used (Table 4). This lack of effect of LUI is apparent in the PCAs (Fig. 3), where LUI is orthogonal (perpendicular) to most diversity indices, indicating that it is independent of them and that there is little overlap in how plots are discriminated based on LUI or diversity. In contrast, we found evidence for effects of LUI on plant diversity for three of the six metrics (S, H’, and D_2_). Furthermore, the magnitude of this effect was similar for all six metrics. We used Bonferroni corrected *P*-values to account for multiple comparisons, but if only one diversity index had been chosen a priori for analysis all but E would have been significantly affected by LUI at the more typical *α* = 0.05.

**Table 4:** Results of linear regression of richness (S), Berger–Parker dominance (BP), Shannon’s diversity (H’), Simpson’s diversity (D_1_), Simpson’s dominance (D_2_), and Simpson’s evenness (E) of various traits measured in grassland plots in and around *Plantago lanceolata* on land use intensity [*F*, *P*, *r*]

	Plant (*N* = 60)	AMF (*N* = 60)	Aboveground arthropods (*N* = 60)	Belowground insect larvae (*N* = 20)	Molecular features (*N* = 60)	Chemical features (*N* = 59)
S	**9.68, 0.0029,** −**0.38**	0.004, 0.947, −0.01	0.08, 0.774, 0.04	0.09, 0.771, 0.07	1.54, 0.220, −0.16	0.69, 0.411, 0.11
BP	7.40, 0.0086, 0.34	0.04, 0.841, 0.03	1.21, 0.276, 0.14	0.001, 0.970, −0.01	2.19, 0.144, 0.19	0.01, 0.918, −0.01
H’	**9.44, 0.0032,** −**0.37**	0.03, 0.872, −0.02	0.55, 0.462, −0.10	0.05, 0.819, 0.05	2.84, 0.097, −0.22	0.16, 0.687, 0.05
D_1_	7.33, 0.0089, −0.33	0.11, 0.742, −0.04	0.85, 0.362, −0.12	0.03, 0.868, 0.04	0.52, 0.475, −0.09	0.005, 0.946, −0.01
D_2_	**8.86, 0.0043,** −**0.36**	0.13, 0.720, −0.05	0.78, 0.380, −0.12	0.002, 0.989, 0.003	0.59, 0.445, −0.10	0.04, 0.842, 0.03
E	1.27, 0.2650, −0.15	0.26, 0.615, −0.07	1.35, 0.251, −0.15	0.06, 0.815, −0.06	0.04, 0.841, −0.03	0.01, 0.929, 0.01

Values in bold indicate significance at Bonferroni corrected *α* of 0.05/6 = 0.0083.

## Discussion

We compared diversities of multiple organism/trait groups across a land use gradient in order to determine how the choice of index affected results of path analyses. We also tested which diversity indices provided the greatest ability to discriminate sites, and whether or not the effect of land use on diversity depended on the diversity index chosen.

### Detecting community dynamics

Our ability to detect relationships between diversities of organisms/traits was clearly influenced by the choice of diversity index, despite the fact that all path models (except that using E) fit the data. The failure of our path model to fit the E data suggests that interactions between diversities in our system are driven primarily by differences in abundance, and not by changes in evenness. Model fit increased slightly as rare species/traits were excluded from the index used, suggesting that rare species/traits were behaving in ways deviating from model predictions. However, similar fit statistics using RMSEA and TLNNFI suggest that any such deviations were small. When using BP and focusing only on the most abundant species/trait, we detected a negative dependence of *P. lanceolata* chemical diversity on herbaceous plant diversity. This shows that as the abundance of the most abundant plant species increases, the abundance of the most abundant chemical metabolite declines. This pattern also holds when using D_1_ and D_2_, in fact with a higher path coefficient, indicating that when other highly abundant species/traits are included, the relationship between plant and chemical diversity is even stronger. When moderately rare species/traits are also considered using H’, even more relationships become apparent. The positive dependence of aboveground arthropod diversity on chemical diversity, and the negative dependence of aboveground arthropod diversity on molecular diversity may therefore be driven equally by rare and abundant species, while abundant species/traits do not seem important for these interactions. The positive dependence of aboveground arthropod diversity on plant diversity is apparent in the models using H’ and S, suggesting that rare species are driving this interaction. The presence of a significant path from plant to chemical diversity for all indices (except E) suggests that changes in both rare and abundant metabolites are negatively affected by changes in rare and abundant plants.

The a priori choice of only one index for a path analysis could have profound consequences on interpretation of relationships between organisms/traits. Running models with a range of diversity indices along Hill’s series allowed us to better understand interactions within our system. Abundance of rare to moderately rare arthropods was positively affected by abundance of rare to moderately rare plants. This may be due to increased niche availability for specialist insect species as plant diversity increased. The negative relationship we observed between plant and chemical diversity for all indices, except E, was also apparent in a separate analysis (only H’ was calculated) using more extensive chemical and plant diversity data sets (T. S. M. Maier & C. M. Müller, unpubl. data). This persistent negative relationship between plant and chemical diversity could be explained by likely reductions in *P. lanceolata* abundance as plant diversity increased and other plant species took up space in the system. In sites with low plant diversity, intraspecific *P. lanceolata* competition could affect chemical composition (Barton and Bowers 2006). Any decreases in *P. lanceolata* abundance associated with increasing plant diversity would also be expected to lead to reduced attack of *P. lanceolata* by specialist herbivores of this plant and therefore reduced induction of defense responses, seen as reduced diversity in the metabolic profile of the plant. Positive relationships between chemical and aboveground arthropod diversity may be explained by increased production of compounds attracting and/or stimulating pollinators, herbivores, and parasitoids of herbivores, and by induction responses of the plant to different interacting species (Sutter and Müller 2011). These further hypothesized interactions between *P. lanceolata* metabolites and different insect groups suggested by the current analysis could be specifically tested in future experiments or field sampling campaigns.

### Verifying earlier findings

As in other studies (Wilsey et al. 2005; Heino et al. 2008), we found that S provided a poor ability to discriminate sites, while the compound diversity measures, primarily D_1_ and D_2_, provided the greatest such ability. The failure of E to effectively discriminate sites shows that the synthesis of richness and abundance information is necessary for site discrimination and that the individual components of the compound diversity measures (S and E) are much less informative when considered independently. The greater ability of measures derived from Hill’s *N*_2_ (D_1_, D_2_) to discriminate sites further suggests that site differences are largely based on differences in abundant species.

A further strength of compound diversity measures over species richness is their reduced dependence on sampling effort (Magurran 2004). We likely underestimated diversity of plants, AMF, belowground insect larvae, and three molecular loci (Table 2). This may be because we sampled multiple grasslands spread across Germany, and our sampling plan may not have been sufficient to adequately catalog the diversity of some organisms/traits across such a broad area. For these groups, compound diversity indices are expected to be more robust than S, although they are still influenced to some extent by sample size (Magurran 2004). In contrast, we may have overestimated chemical diversity, as number of peaks is probably higher than the number of real metabolites due to fragments, adducts, and isotopes that may occur in the metabolic fingerprinting approach. On the other hand, metabolic fingerprinting was only done of polar metabolites, so the overall metabolite number in each sample is in total again higher.

Diversity of organisms/traits in our system was remarkably uninfluenced by land use changes including increased fertilization, grazing, and mowing. We found no effect of land use on diversities of AMF, aboveground arthropods, belowground insect larvae, or *P. lanceolata* molecular or chemical diversity. Aboveground arthropods were identified to order, while belowground insect larvae were identified to family. It is not entirely clear what level of resolution is achieved with the NS31-AM1 primers used in the AMF analysis, but it is almost certainly higher than species level. Any effects of LUI may only be apparent at finer taxonomic scales. In this analysis, we focused on species associated with *P. lanceolata*, and it is possible that effects of LUI would be observed in broader communities. At least in this system, molecular and chemical diversity were less sensitive to land use than herbaceous plant species. Three of the six plant diversity measures (S, H’, D_2_) were negatively affected by LUI. The differing sensitivities of diversity indices to LUI in our analysis were largely driven by our need to correct for multiple comparisons. In analyses using only one diversity index, similar significant effects of LUI would have been detected using any of the indices we included, except E. Thus, when conducting simple statistical analyses of a specific effect of disturbance on diversity, the choice of index does not appear particularly important.

### Trait-based diversity measures

We included two trait-based measures of diversity in order to assess their performance relative to species diversity. Chemical and plant diversity (except evenness) were consistently negatively correlated, but there were no other correlations between chemical and molecular diversity and diversity of any other organism. This shows that trait-based diversity measures can capture unique information and can be useful when considered along with species diversity. For example, based on the significant relationships identified in our path analysis between chemical, plant, and arthropod diversity, we were able to formulate new hypotheses about regulation of defense induction in our system that would not have been apparent without chemical diversity in the model.

As for the measures of species diversity, the compound diversity indices (D_1_, D_2_, H’) outperformed others when differentiating sites based on molecular and chemical diversity data. This suggests that, at least very generally, relationships between richness and evenness of these traits are similar to those seen in species diversity. Also, estimates of total richness were much closer to observed richness values for molecular and chemical diversity than for most organism groups. This suggests that it is easier to thoroughly sample at least some traits than it is to sample species. Given the dependence of many indices on sampling effort, this is a clear benefit of trait-based diversity measures. Overall, the trait-based measures of diversity performed very well and potentially have a place in other biodiversity studies as thorough measures of richness that capture information largely missed by organismal species diversity.

## Conclusions

The importance of carefully deciding how to quantify diversity in multiple organism/trait groups is apparent from our analysis. The failure of any species/trait group other than herbaceous plants to detect effects of land use also calls into question the practice of using easy to measure indicator taxa to estimate effects on other taxa. At the very least, analyses such as this should precede selection of such indicator taxa to ensure that nonindicator taxa are in fact behaving as expected.

We could not identify one ideal diversity index. Simpson’s indices, D_1_ and D_2_, performed best when differentiating sites, but simpler indices were slightly preferable when detecting effects of land use intensity on diversity. All indices except E were equally effective when fitting path models to describe relationships between organisms/traits, although the greatest number of relationships was apparent when using H’. We assessed performance of each index largely as the significance of effects or number of relationships detected, with the inherent assumption that such effects and relationships did in fact exist. If effects of LUI or relationships between organism/trait groups are not strong, indices that did not detect effects may more accurately represent reality. Modeling approaches using artificial systems where relationships are predefined could help resolve this issue. While analyses of synthetic data would allow one to completely control community structure and avoid biases related to varying sample sizes, such an approach would also disallow the ecological realism obtained in the present analysis. It is clear that relationships between diversity indices do not always follow mathematically predicted patterns (Stirling and Wilsey 2001; Nagendra 2002), and it is therefore important to perform analyses such as these on real data to ensure that conclusions will be valid in the field.

Other attempts to identify an ideal diversity measure have failed to find one, and instead suggest reporting at least two measures (Whittaker 1972; Stirling and Wilsey 2001; Heino et al. 2008). Including multiple diversity measures, spread along Hill’s continuum (Hill 1973), provided us with a more complete understanding of how shifts in rare and abundant species were driving interactions. Additional benefits of using the Hill series instead of the closely related more traditional indices include the simplified interpretation of results because units are always in effective number of species regardless of the position along the series (Jost 2006). Furthermore, effective species numbers behave as one would intuitively expect when diversity is doubled or halved, while other standard indices of diversity (H’, D_1_, D_2_) do not (Jost 2006).

### Recommendations for implementation

Data mining to identify an index providing strong significant effects should be discouraged. We advocate a priori selection of, at most, a small number of diversity measures along Hill’s series that are expected to capture the important aspects of diversity in the system under study. If effects are expected to be more apparent in rare species/traits, then S would be appropriate. However, if dominant species/traits are expected to be more important, then D_1_, D_2,_ and BP would be more appropriate. H’ could be used in situations where rare and abundant species/traits are expected to be equally important. Comparison of a few carefully chosen indices could greatly enhance understanding of the complex components driving diversity.

## Data Accessibility

R scripts needed to complete the analyses described here, along with sample data sets, can be found in the supplementary information.

## Supporting Information

Data S1 R script file containing all code necessary to run the analyses described here.

Table S1 Example land use intensity (LUI) data.

Table S2 Example species abundance data for hypothetical organism 1.

Table S3 Example species abundance data for hypothetical organism 2.

Table S4 Example species abundance data for hypothetical organism 3.

Table S5 Example species abundance data for hypothetical organism 4.

Table S6 Example species abundance data for hypothetical organism 5.

Table S7 Example species abundance data for hypothetical organism 6.

**Appendix S1**Methods. **Appendix Table S1.** Correlations between richness (S), Berger-Parker dominance (BP), Shannon’s diversity (H’), Simpson’s diversity (D_1_), Simpson’s dominance (D_2_), and Simpson’s evenness (E) for each organism/character group (*P*, *r*) measured in grassland plots in and around *Plantago lanceolata*.
