# Supplementary material for: Choosing and using diversity indices: insights for ecological applications from the German Biodiversity Exploratories
Source: Ecol Evol. 2014 Aug 28;4(18):3514–24. doi: 10.1002/ece3.1155 (PMC4224527; doi:10.1002/ece3.1155)
Supplement: Supplementary file 9 — Appendix S1Methods. Appendix Table S1. Correlations between richness (S), Berger-Parker dominance (BP), Shannon’s diversity (H’), Simpson’s diversity (D1), Simpson’s dominance (D2), and Simpson’s evenness (E) for each organism/character group (P, r) measured in grassland plots in and around Plantago lanceolata. [file ece30004-3514-sd9.doc]

SUPPLEMENTARY METHODS

*Sites sampled*

In Schorfheide Chorin we sampled 15 sites; SEG06, SEG08, SEG31, SEG32, SEG33, SEG34, SEG35, SEG39, SEG40, SEG41, SEG43, SEG44, SEG45, SEG46, and SEG47. Similarly, 15 sites were sampled in Hainich Dün; HEG06, HEG08, HEG09, HEG11, HEG17, HEG18, HEG20, HEG30, HEG34, HEG36, HEG42, HEG43, HEG44, HEG46, and HEG48. Coverage was more extensive in Schwäbische Alb, with 30 sites covered; AEG02, AEG03, AEG06, AEG07, AEG09, AEG11, AEG12, AEG13, AEG15, AEG17, AEG18, AEG21, AEG22, AEG25, AEG26, AEG27, AEG28, AEG30, AEG31, AEG32, AEG33, AEG34, AEG36, AEG38, AEG40, AEG41, AEG42, AEG43, AEG47, and AEG49. Detailed site descriptions can be found in Fischer et al., (2010).

*Plant diversity*

We recorded plant species diversity by identifying herbaceous plant species and estimating the percentage cover of each species in a 15 cm radius around all 10 focal *P. lanceolata* plants in each plot.

*Arbuscular mycorrhizal fungal diversity*

Arbuscular mycorrhizal fungal diversity was assessed using terminal restriction fragment length polymorphism (T-RFLP) analysis. A 10 cm diameter soil core (0 – 10 cm depth) from immediately beneath a focal *P. lanceolata* plant in each site was collected between July and August 2008 and stored at -20 °C until analysis. Cores were thawed overnight at 4 °C, then split vertically down the center so that a soil sample (~ 3 g) could be collected from the *P. lanceolata* rhizosphere. These samples were again stored at -20 °C until DNA was extracted from 250 mg soil with a MoBio PowerSoil DNA Extraction Kit (96well) kit (Carlsbad, CA, USA), and AMF DNA was amplified with a nested PCR approach. We began with GLOMER WT0/GLOMER 1536 primers (0.5 μM each), 1X FIREPol 5xPCR Mix, 7.5mM MgSO_4_ (Solis BioDyne, Tartu, Estonia), and 1 μL of template DNA in a final volume of 50 μL (Wubet et al. 2006). The PCR conditions were 98 °C for 30 sec, followed by 5 cycles of 94 °C for 30 sec, 60 °C for 45 sec, and 72 °C for 1 min with the 60 °C step decreasing by 1 C each cycle; then 25 cycles of 94 °C for 30 sec, 55 °C for 30 sec, 72 °C for 1 min, and finally 72 °C for 5 min in an Eppendorf thermocycler (Eppendorf, Hamburg, Germany). We performed two separate reactions for the second PCR using NS31-FAM and one of two AM1 primers: the original (Helgason et al. 1998), and a modified primer designed to capture more AMF genera than the original AM1 primer (AM1b: 5’-  [CTT TGG TTT CCC ATA RGG TGC C](https://ecom.mwgdna.com/services/webgist/mops.tcl?oligoSequence=CTT TGG TTT CCC ATA AGG CGC C&ot=OLIGO_DNA&oligo_name=cmt_&action=properties) -3’) (unpublished, Wubet). The second PCR was performed with the same recipe as the first PCR, but with PCR conditions of 98 °C for 1 min, 30 cycle of 94 °C for 30 sec, 63 °C for 30 sec, 72 °C for 1 min, followed by 72 °C for 5 min. PCR products were cleaned with a Nucleospin ExtractII kit (Machery Nagel, Düren, Germany), before quantification of DNA with a NanoPhotometer (Implen, Munich). We then combined 40 ng DNA from each AM1 primer reaction to regenerate complete samples. We used *HinfI* (Fermentas, St. Leon-Rot, Germany) in digests of 80 ng DNA, 2 μL buffer, and 0.3 units enzyme in 20 μL total volume. Digestions were incubated at 37 °C for 2 hours, then cleaned with a Nucleoseq kit (Machery Nagel, Düren, Germany) before analysis on an ABI 3730xl Genetic Analyzer with a custom made ROX size standard (BioVentures,

Murfreesboro, TN, USA).

Terminal restriction fragment (TRF) sizes and peak heights were determined using GeneMapper 3.7 software (Applied Biosystems, Carlsbad, CA, USA) with a threshold of 75 AU. Total fluorescence of each profile was standardized (Dunbar et al. 2001), then TRFs were aligned with T-REX (http://trex.biohpc.org/index.aspx) to combine fragments lengths differing by less than one base pair into the same TRF (Culman et al. 2009). TRF number was used as a surrogate for species richness, and TRF peak height as a surrogate for species abundance. The latter may not always be accurate due to the existence of the same TRF across different AMF species, PCR bias during amplification, and differences in gene copy number among different AMF (Corradi et al. 2007). However, the technique had been used to calculate diversity indices for AMF (Burke 2008), and it is instructive to do so in this context to allow for comparisons with diversity indices of other organism and character groups investigated in this study, while bearing in mind that for AMF specifically, richness remains the most reliable metric.

*Above ground arthropod diversity*

We used a modified hand-held vacuum cleaner (Rowenta AC 8818 Silencer 12V, Offenbach/Main, **SEB Deutschland GmbH)** to collect all arthropods resting on all 10 *P. lanceolata* plants in each plot. We began vacuuming over the inflorescences of each plant, moved down to the rosette, and then back up in order to prevent escape of the arthropods. Afterwards the arthropods were transferred with a small paintbrush to 70% ethanol and stored in the lab until individuals were identified to order level.

*Below ground insect larval diversity*

Only 20 sites were sampled for below ground insect larval diversity; SEG06, SEG08, SEG33, SEG34, SEG35, HEG06, HEG08, HEG11, HEG30, HEG48, AEG02, AEG03, AEG06, AEG07, AEG09, AEG11, AEG13, AEG22, AEG26, and AEG27.

Three soil cores of 5 cm diameter and 10 cm depth were taken randomly from a 1 m x 6 m subplot at each site in September/October 2009. Visible insect larvae were sorted out by hand and smaller larvae were extracted by subsequent heat extraction (MacFayden 1961). Larvae were stored in 70 % ethanol until identification to family level.

*Plantago molecular diversity*

To assess molecular diversity we sampled between five and eight individuals of *P. lanceolata* and extracted DNA from 50 mg dried leaf tissue. The tissue was homogenized with a mixer mill and DNA was extracted with a commercial extraction kit (NucleoSpin96 Plant, Macherey Nagel, Düren, Germany). The DNA content was measured with a fluorescence spectrometer and each DNA sample was diluted to 5 ng per 5 µl with nuclease free water.

We used the 5 polymorphic, dinucleotid, nuclear microsatellite loci (simple sequence repeats) developed by Hale and Wolff (2003). These microsatellite markers were labeled with fluorescent dye and multiplexed for PCR amplification. PCR reactions were carried out in 10 µl volumes containing 5 ng DNA, 5µl Qiagen HotStarTaq Master Mix Kit (Valencia, USA, consisting of multiplex PCR buffer with a final concentration of 3 mM MgCl_2_, dNTP mix, and HotStarTaq DNA polymerase), 0.5 µl of locus-specific 5’ fluorescent labeled forward primer (6-FAM ^TM^, HEX ^TM^: Microsynth, Balgach, Switzerland, and NED ^TM^ Applied Biosystems), and non-labeled reverse primer. PCR amplification was performed in a 96-well PTC-100 TM Programmable Thermal Controller (MJ Research, Inc.,Waltham, USA) by using the following cycling protocol: 15 min at 95°C; 30 cycles consisting of 30 sec at 52°C, 30 sec at 72 °C, 30 sec 95°C, followed by 1min at 52°C and 8 min at 72°C. Fluorescent PCR fragments were visualized by capillary electrophoresis on an ABI PRISM® 310 Genetic Analyzer (Applied Biosystems) with GeneScan 500 ROX as a standard and analyzed by the GeneMapper® Analysis Software version 3.7 (Applied Biosystems). To ensure that the multiplexing had no effect on the size of the amplified DNA fragments, loci were amplified singly for several individuals and compared with the multiplex profiles. Furthermore, we re-analyzed several samples to test the reproducibility of band length and genotyping. We then assessed the number and frequencies of alleles at each locus, calculated the diversity indices for each locus separately and averaged the values across loci.

*Plantago chemical diversity*

Site HEG11 was not sampled for *P. lanceolata* chemical diversity. In May 2009, the oldest and the youngest leaf of one *P. lanceolata* plant per plot were cut and transferred to methanol:dichloromethane (2:1, pH 6). The sample was homogenized and stored at 4°C for one week. Then leaves were extracted in the laboratory three times at different pHs (pH 6, pH 2, pH 9) following the protocol by Maier et al., (2010). Supernatants from each extraction step were pooled and water was added (2:1:1 methanol:dichloromethane:water) for phase separation. The aqueous phases were stored in Eppendorff tubes at 4 °C until analyses. Remaining pellets were dried for determination of dry weight (Sartorius LA120S, precision +/- 0.1 mg, Göttingen, Germany).

Metabolic fingerprinting was performed on an UHPLC-TOF-MS (1290 Infinity UHPLC and 6210 TOF, Agilent Technologies, Santa Clara, U. S. A.) equipped with a Grom-Sil 120 ODS-4-HE-column (150 x 2 mm, 3 µm; Alltech Grom GmbH, Rottenburg-Hailﬁngen, Germany) using a gradient from water with 0.1% formic acid (98-100%, Co. Merck, Darmstadt, Germany; solvent A) to acetonitrile (LC-MS grade, Co. Fisher Scientiﬁc UK Limited, Loughborough, Great Britain; solvent B) with 0.1% formic acid at a ﬂow of 0.75 ml and an oven temperature of 35 °C. The gradient started at 5% solvent B with a hold for 1 min, increased from 2-16 min to 95% B and was held for 1 min at 95% B, followed by a cleaning and column equilibration cycle. Measurements were done in positive mode with a Dual ESI source (gas temperature: 350 °C, drying gas ﬂow: 11 l/min, nebulizer pressure: 55 psig). For further details see Maier et al., (2010).

Metabolite data were pre-processed with R (version 2.9.0 and newer) package “xcms“ (Smi*th et a*l. 2006; R Development Core Team 2008; Tautenha*hn et a*l. 2008) (method = ”centWave”, ppm = 23, profmethod = ”bin”, peakwidth = c(5,12), snthresh = 10, prefilter = c(3,200), fitgauss = T; settings for ”group”: bw = 30, minfrac = 0.5, minsamp = 1, mzwid = 1, max = 50, sleep = 0). Peaks occurring in blanks were subtracted from samples, except peaks with mean intensities being 100 or more times intensive than in blanks. Intensities lower than 1 ･ 10^3^ were not considered. Each peak was considered a metabolite, for simplicity. Data were log transformed for normalization.

**Appendix Table S1.** Correlations between richness (S), Berger-Parker dominance (BP), Shannon’s diversity (H’), Simpson’s diversity (D_1_), Simpson’s dominance (D_2_), and Simpson’s evenness (E) for each organism/character group (*P*, *r*) measured in grassland plots in and around *Plantago lanceolata*. All statistics refer to within group correlations. Values in bold indicate significance at Bonferroni corrected α of 0.05/15 = 0.0033.

| Plant (*N* = 60) | BP | H’ | D_1_ | D_2_ | E |
| --- | --- | --- | --- | --- | --- |
| S | **<0.0001,**  **-0.80** | **<0.0001,**  **0.94** | **<0.0001,**  **0.86** | **<0.0001,**  **0.89** | 0.7993,  -0.03 |
| BP |  | **<0.0001,**  **-0.92** | **<0.0001,**  **-0.97** | **<0.0001,**  **-0.97** | **<0.0001,**  **-0.48** |
| H’ |  |  | **<0.0001,**  **0.98** | **<0.0001,**  **0.98** | 0.0490,  0.26 |
| D_1_ |  |  |  | **<0.0001,**  **0.99** | **0.0018,**  **0.40** |
| D_2_ |  |  |  |  | **0.0020,**  **0.39** |
| Arbuscular mycorrhizal fungi (*N* = 60) | BP | H’ | D_1_ | D_2_ | E |
| S | **<0.0001,**  **-0.72** | **<0.0001,**  **0.95** | **<0.0001**  **0.88** | **<0.0001,**  **0.87** | 0.2102,  0.16 |
| BP |  | **<0.0001,**  **-0.88** | **<0.0001,**  **-0.94** | **<0.0001,**  **-0.93** | **<0.0001,**  **-0.76** |
| H’ |  |  | **<0.0001,**  **0.98** | **<0.0001,**  **0.97** | **0.0002,**  **0.47** |
| D_1_ |  |  |  | **<0.0001,**  **0.99** | **<0.0001,**  **0.61** |
| D_2_ |  |  |  |  | **<0.0001,**  **0.59** |
| Aboveground arthropod (*N* = 60) | BP | H’ | D_1_ | D_2_ | E |
| S | 0.1218,  -0.20 | **<0.0001,**  **0.59** | 0.0037,  0.37 | **0.00314,**  **0.38** | **<0.0001,**  **-0.56** |
| BP |  | **<0.0001,**  **-0.80** | **<0.0001,**  **-0.92** | **<0.0001,**  **-0.92** | **<0.0001,**  **-0.61** |
| H’ |  |  | **<0.0001,**  **0.95** | **<0.0001,**  **0.96** | 0.0421,  0.26 |
| D_1_ |  |  |  | **0.0001,**  **>0.99** | **<0.0001,**  **0.50** |
| D_2_ |  |  |  |  | **<0.0001,**  **0.50** |
| Belowground insect larvae (*N* = 20) | BP | H’ | D_1_ | D_2_ | E |
| S | **0.0005,**  **-0.70** | **<0.0001,**  **0.98** | **<0.0001,**  **0.90** | **<0.0001,**  **0.92** | 0.3735,  -0.21 |
| BP |  | **<0.0001,**  **-0.81** | **<0.0001,**  **-0.92** | **<0.0001,**  **-0.90** | 0.0219,  -0.51 |
| H’ |  |  | **<0.0001,**  **0.96** | **<0.0001,**  **0.96** | 0.8820,  -0.04 |
| D_1_ |  |  |  | **<0.0001,**  **0.97** | 0.3374,  0.23 |
| D_2_ |  |  |  |  | 0.5616,  0.14 |
| *Plantago* molecular features (*N* = 60) | BP | H’ | D_1_ | D_2_ | E |
| S | **<0.0001**  **-0.56** | **<0.0001,**  **0.87** | **<0.0001,**  **0.59** | **<0.0001,**  **0.59** | 0.1462,  0.19 |
| BP |  | **<0.0001,**  **-0.56** | **<0.0001,**  **-0.88** | **<0.0001,**  **-0.84** | **<0.0001,**  **-0.73** |
| H’ |  |  | **<0.0001,**  **0.59** | **<0.0001,**  **0.60** | 0.0399,  -0.27 |
| D_1_ |  |  |  | **<0.0001,**  **0.95** | **<0.0001,**  **0.87** |
| D_2_ |  |  |  |  | **<0.0001,**  **0.90** |
| *Plantago* chemical features (*N* = 59) | BP | H’ | D_1_ | D_2_ | E |
| S | 0.0210,  -0.30 | **<0.0001,**  **0.79** | **<0.0001,**  **0.61** | **<0.0001,**  **0.60** | **<0.0001,**  **0.52** |
| BP |  | **<0.0001,**  **-0.634** | **<0.0001,**  **-0.86** | **<0.0001,**  **-0.88** | **<0.0001,**  **-0.90** |
| H’ |  |  | **<0.0001,**  **0.92** | **<0.0001,**  **0.91** | **<0.0001,**  **0.88** |
| D_1_ |  |  |  | **<0.0001,**  **0.99** | **<0.0001,**  **0.99** |
| D_2_ |  |  |  |  | **<0.0001,**  **0.99** |

REFERENCES

Burke, D. J. (2008) Effects of *Alliaria petiolata* (garlic mustard; Brassicaceae) on mycorrhizal colonization and community structure in three herbaceous plants in a mixed deciduous forest. *American Journal of Botany,* **95,** 1416-1425.

Corradi, N., Croll, D., Colard, A., Kuhn, G., Ehinger, M. & Sanders, I. R. (2007) Gene copy number polymorphisms in an arbuscular mycorrhizal fungal population. *Applied and Environmental Microbiology,* **73,** 366-369.

Culman, S. W., Bukowski, R., Gauch, H. G., Cadillo-Quiroz, H. & Buckley, D. H. (2009) T-REX: Software for the processing and analysis of T-RFLP data. *BMC Bioinformatics,* **10,** 171.

Dunbar, J., Ticknor, L. O. & Kuske, C. R. (2001) Phylogenetic specificity and reproducibility and new method for analysis of terminal restriction fragment profiles of 16S rRNA genes from bacterial communities. *Applied and Environmental Microbiology,* **67,** 190-197.

Fischer, M., Bossdorf, O., Gockel, S., Hänsel, F., Hemp, A., Hessenmöller, D., Korte, G., Nieschulye, J., Pfeiffer, S., Prati, D., Renner, S., Schöning, I., Schumacher, U., Wells, K., Buscot, F., Kalko, E. K. V., Linsenmair, K. E., Schulze, E.-D. & Weisser, W. W. (2010) Implementing large-scale and long-term functional biodiversity research: The Biodiversity Exploratories. *Basic and Applied Ecology,* **11,** 473-485.

Hale, M. L. & Wolff, K. (2003) Polymorphic microsatellite loci in *Plantago lanceolata*. *Molecular Ecology Notes,* **3,** 134-135.

Helgason, T., Daniell, T. J., Husband, R., Fitter, A. H. & Young, J. P. W. (1998) Ploughing up the wood-wide web? *Nature,* **394,** 431.

MacFayden, A. (1961) Improved funnel-type extractor for soil arthropods. *Journal of Animal Ecology,* **30,** 171-184.

Maier, T. S., Kuhn, J. & Müller, C. (2010) Proposal for field sampling of plants and processing in the lab for environmental metabolic fingerprinting. *Plant Methods,* **6,** 6.

R Development Core Team (2008) R: A language and environment for statistical computing. (ed^(eds. R Foundation for Statistical Computing, Vienna, Austria.

Smith, C. A., Want, E. J., O'Maille, G., Abagyan, R. & Siuzdak, G. (2006) XCMS: Processing mass spectrometry data for metabolite profiling using nonlinear peak alignment, matching and identification. *Analytical Chemistry (Washington),* **78,** 779-787.

Tautenhahn, R., Bottcher, C. & Neumann, S. (2008) Highly sensitive feature detection for high resolution LC/MS. *BMC Bioinformatics,* **9,** 504.

Wubet, T., Weiß, M., Kottke, I., Teketay, D. & Oberwinkler, F. (2006) Phylogenetic analysis of nuclear small subunit rDNA sequences suggests that the endangered African Pencil Cedar, *Juniperus procera*, is associated with distinct members of *Glomeraceae*. *Mycological Research,* **110,** 1059-1069.
